# Use and Perceived Impact of Simulation-Based Training in Surgical Education: A Multinational Cross-Sectional Study

**DOI:** 10.7759/cureus.117725

**Published:** 2026-10-10

**Authors:** Nishat Jahan Chowdhury, Erica Khandaker, Tushar Kumar Sarker, Laila Arjuman, Shumaiya Ferdaus, Atashi Irtiza, Md. Adib Siddiki

**Affiliations:** 1 Breast Surgery, Sylhet Women's Medical College Hospital, Sylhet, BGD; 2 Surgery, Sylhet Women's Medical College Hospital, Sylhet, BGD; 3 General Surgery, Shaheed Suhrawardy Medical College and Hospital, Dhaka, BGD; 4 Hepatology, Rajshahi Medical College, Rajshahi, BGD; 5 Anatomy, Sylhet Women's Medical College Hospital, Sylhet, BGD; 6 Cardiovascular Surgery, Bangladesh Medical University, Dhaka, BGD; 7 General Surgery, Holy Family Red Crescent Medical College and Hospital, Dhaka, BGD

**Keywords:** medical education, multinational study, simulation-based training, surgical education, surgical trainees

## Abstract

Importance: Simulation-based surgical training is increasingly adopted as a method to enhance surgical skill development while reducing the risk of harm to patients.

Objective: This study aims to evaluate the level of exposure to, experiences with, and attitudes toward simulation-based surgical training among healthcare professionals from different countries.

Design, setting, and participants: A cross-sectional survey was conducted between January 6 and January 25, 2026, in 49 hospitals located in Bangladesh, the United Kingdom, the Maldives, Syria, and India. The study included 112 participants comprising medical students, interns, postgraduate trainees, medical officers, and consultants.

Main outcomes and measures: Simulation training exposure, modalities used, skills practiced, session frequency and duration, and perceived benefits.

Results: Fifty-nine participants (52.7%) reported prior experience with simulation-based training. Bench-top trainers and cadaveric models were the most frequently used modalities, and suturing was the skill most commonly practiced. Most training sessions were conducted once annually and lasted less than two hours. A high proportion of participants reported improved understanding of procedural steps, increased confidence, and greater preparedness for clinical practice following simulation training.

Conclusions and relevance: Simulation-based surgical training is utilized across a wide range of clinical settings and provides a valuable supplement to conventional surgical education, supporting skill development, confidence, and preparedness across diverse training environments.

## Introduction

Developing competent surgical professionals is a critical priority for healthcare systems worldwide, as surgical care constitutes a substantial component of modern healthcare delivery and patient outcomes [1]. With increasing surgical complexity, rapid technological advancements, and growing expectations for patient safety, the demands placed on surgical training programs have expanded considerably. Traditionally, surgical education in many countries has relied on an apprenticeship-based model that combines classroom instruction, bedside teaching, and supervised participation in operative procedures [2]. While this approach allows trainees to gain experience directly under the guidance of experienced surgeons, it also presents several challenges.

Clinical case exposure, for example, can vary considerably among trainees depending on institutional resources, patient volumes, and case availability, potentially leading to inconsistencies in skill acquisition. In addition, ethical concerns surrounding the acquisition of technical skills through practice on live patients have gained increasing attention in discussions of patient safety and quality of care [2,3]. Furthermore, the traditional apprenticeship model may offer limited opportunities for deliberate and repeated practice of specific procedural skills, which are essential for achieving surgical competence [3]. In response to these challenges, medical education has increasingly moved toward competency-based training frameworks that emphasize measurable skill acquisition, structured assessment, and safe learning environments.

Within this evolving educational landscape, simulation-based education has emerged as a valuable strategy to complement traditional training methods. By providing controlled environments that allow repeated practice and structured feedback, simulation enables trainees to develop both technical and non-technical skills, including decision-making, teamwork, and procedural competence [4,5]. Previous research has demonstrated that simulation-based mastery learning can improve operative performance and may even contribute to improved patient outcomes [6-8].

Despite these benefits, simulation-based training remains underutilized in many low- and middle-income countries due to limitations in resources, infrastructure, and trained faculty [2,8]. In countries such as Bangladesh, surgical training programs often face additional constraints, including high patient loads, limited availability of simulation laboratories, and restricted time for structured skills training. As a result, many institutions continue to rely heavily on traditional apprenticeship-based learning within busy clinical environments, which may reduce opportunities for deliberate practice and structured feedback. Financial limitations and limited institutional investment may further hinder the establishment and sustainability of simulation facilities [9].

Although previous studies have demonstrated the educational effectiveness of simulation-based training [4,5], relatively little empirical evidence exists describing how such training is implemented in routine clinical training environments across diverse healthcare systems. In particular, there is limited information regarding trainees’ exposure to different simulation modalities, the types of procedural skills practiced, the frequency and duration of training sessions, and healthcare professionals’ perceptions of the educational impact of simulation-based training. Understanding these aspects is important for identifying current training practices and informing strategies to strengthen simulation-based surgical education. Therefore, this study aimed to assess exposure to simulation-based surgical training, characterize its use, and evaluate its perceived educational impact - specifically perceived procedural understanding, hand-eye coordination, confidence and preparedness, perceived transferability of skills to clinical practice, attitudes toward the integration of simulation into surgical curricula, and perceived barriers to its implementation - among healthcare professionals from multiple healthcare institutions in a multinational sample.

## Materials and methods

Design, setting, and participants

This quantitative cross-sectional questionnaire-based study was conducted across 49 hospitals in five countries (Bangladesh, the United Kingdom, Maldives, Syria, and India). Participants included undergraduate medical students, surgical interns, medical officers, postgraduate trainees, consultants, and other physicians involved in surgical care.

A total of 112 participants were recruited using convenience sampling, and data were collected using a structured questionnaire administered to eligible participants across participating centers. The questionnaire was distributed electronically, and participation was voluntary. Doctors specializing exclusively in non-surgical subspecialties and participants who declined participation were excluded from the study. Responses were collected using a 5-point Likert scale. All responses were anonymized at the time of collection to ensure confidentiality. Data collection was conducted from January 06, 2026, to January 25, 2026, after which responses were collated and analyzed. Of the 112 participants, institutional affiliation indicated that the majority were based in Bangladesh (n = 88), with smaller numbers from the United Kingdom (n = 6), India (n = 2), Syria (n = 1), and the Maldives (n = 1); institutional affiliation was not specified by 14 participants.

Questionnaire development and pilot testing

The questionnaire was designed by the investigators based on a review of existing literature on simulation-based surgical education [1,3-5] and clinical experience in surgical practice. Prior to full deployment, the questionnaire was piloted among a subset of individuals representative of the intended study population to evaluate clarity and content relevance. Revisions were made in response to pilot feedback, and pilot responses were excluded from the final dataset. The full questionnaire is provided in the Appendices.

Outcomes and variables

The primary outcome was the participant's overall opinion as assessed by responses to the structured questionnaire. A composite score was calculated by summing individual item scores, with higher scores indicating greater overall proficiency.

Ethical consideration

This study involved an anonymous, voluntary questionnaire and did not collect any personal identifiable information. In accordance with institutional and local guidelines for minimal-risk survey research, formal ethical review was not required. Informed consent was implied by voluntary participation in the study. All procedures were conducted in accordance with the principles of the Declaration of Helsinki.

Statistical analysis

Descriptive statistics were used to summarize participant characteristics and questionnaire responses. Continuous variables were reported as means with standard deviations or medians with interquartile ranges, as appropriate, while categorical variables were presented as percentages. For items permitting multiple selections (simulation modality and skills practiced), percentages were calculated using the total number of respondents (n = 112). The item assessing previous exposure to simulation-based learning and the subsequent items on simulation modalities and skills practiced were structured as independent questions rather than as a skip-logic sequence; all participants, regardless of their response to the previous-exposure item, were able to respond to the modality and skills items.

## Results

Study population

A total of 112 individuals were included in the study. Most were medical officers (n = 37, 33.0%), followed by postgraduate trainees (n = 32, 28.6%), interns (n = 21, 18.8%), consultants (n = 11, 9.8%), others (e.g., research fellows, non-training doctors) (n = 8, 7.1%), and undergraduate medical students (n = 3, 2.7%). More than half (n = 59, 52.7%) had <1 year of training in surgery, while 34 (30.4%) had 1-2 years of training, 8 (7.1%) had 3-5 years, and 11 (9.8%) had >5 years of training. Participants represented multiple specialties, including general surgery (n = 48, 42.9%), ophthalmology (n = 18, 16.1%), orthopedics (n = 12, 10.7%), and other combined specialties (n = 34, 30.3%) (Table 1).

**Table 1:** Participant Characteristics (N = 112).

Category	Subcategory	Frequency (n)	Percentage (%)
Current Role	Medical Officer	37	33
	Postgraduate Trainee	32	28.6
	Intern	21	18.8
	Consultant	11	9.8
	Others	8	7.1
	Undergraduate Medical Student	3	2.7
Years of Training	<1 year	59	52.7
	1–2 years	34	30.4
	3–5 years	8	7.1
	>5 years	11	9.8
Primary Specialty	General Surgery	48	42.9
	Ophthalmology	18	16.1
	Orthopedics	12	10.7
	Others	34	30.3
Simulation Exposure	Previous Exposure	59	52.7
	No Previous Exposure	53	47.3

Exposure to simulation-based training

A total of 59 (52.7%) respondents reported prior exposure to simulation-based training, while 53 (47.3%) had not.

The most commonly used simulation modalities were bench-top models (n = 61, 54.5%) and cadaveric models (n = 60, 53.6%). These were followed by virtual reality simulators (n = 36, 32.1%), hybrid simulators (n = 30, 26.8%), and live animal models (n = 14, 12.5%).

Frequently practiced skills included suturing (n = 98, 87.5%), excision techniques (n = 64, 57.1%), injection techniques (n = 58, 51.8%), and cardiopulmonary resuscitation (n = 55, 49.1%). Additional skills included excision of soft tissue swellings (n = 30, 26.8%) and biopsy techniques (n = 29, 25.9%). Advanced procedural skills included laparoscopy (n = 21, 18.8%), flap surgery (n = 17, 15.2%), and endoscopy (n = 9, 8.0%).

Simulation sessions were conducted once a year in 42 participants (37.5%), several times a year in 34 (30.4%), several times a month in 23 (20.5%), several times a week in 12 (10.7%), and daily in 1 (0.9%). Typical session duration was <1 hour in 50 respondents (44.6%), 1-2 hours in 44 (39.3%), and >2 hours in 18 (16.1%).

The primary objective of simulation training was skill acquisition (n = 92, 82.1%), followed by assessment (n = 8, 7.1%), revision (preclinical) (n = 8, 7.1%), and emergency preparedness (n = 4, 3.6%) (Table 2).

**Table 2:** Exposure to Simulation-Based Surgical Training Among Participants (N = 112). *Participants could select more than one response; therefore, percentages may not total 100%.

Characteristic	Category	n	%
Simulation modality*	Bench-top models	61	54.5
	Cadaveric models	60	53.6
	Virtual reality simulators	36	32.1
	Hybrid simulators	30	26.8
	Live animal models	14	12.5
Skills practiced*	Suturing	98	87.5
	Excision techniques	64	57.1
	Injection techniques	58	51.8
	Cardiopulmonary resuscitation	55	49.1
	Swelling excision	30	26.8
	Biopsy techniques	29	25.9
	Laparoscopy	21	18.8
	Flap surgery	17	15.2
	Endoscopy	9	8
Frequency of simulation sessions	Once per year	42	37.5
	Several times per year	34	30.4
	Several times per month	23	20.5
	Several times per week	12	10.7
	Daily	1	0.9
Typical session duration	<1 hour	50	44.6
	1–2 hours	44	39.3
	>2 hours	18	16.1
Primary objective of simulation	Skill acquisition	92	82.1
	Assessment	8	7.1
	Revision (preclinical)	8	7.1
	Emergency preparedness	4	3.6

Simulation-based training was widely perceived to enhance understanding of procedural steps, with 91 individuals (81.3%) expressing agreement or strong agreement. An improved understanding of surgical anatomy was reported by 86 (76.8%), while 90 (80.4%) noted better hand-eye coordination.

A reduced dependence on direct supervision was noted by 77 (68.8%), and 74 (66.1%) indicated an improved ability to anticipate intraoperative complications. A high proportion agreed that skills acquired through simulation were transferable to real surgical practice, as reported by 88 respondents (78.6%).

Enhanced transition into the operating theater was reported by 91 (81.3%), alongside reduced early clinical errors in 90 (80.4%). Reduced fear of making mistakes on real patients was indicated by 91 (81.3%), while improved mental preparedness before assisting or performing procedures was observed in 94 responses (83.9%).

Simulation-based training was perceived to promote a safe learning environment by 95 (84.8%), with 89 (79.5%) also reporting improvements in confidence and self-esteem (Table 3, Figure 1).

**Table 3:** Perceived Impact of Simulation-Based Learning on Skill Development (N = 112).

Item	Mean	SD	Agree (%)
Understanding procedural steps more clearly	4.29	1	81.2
Understanding surgical anatomy more clearly	4.21	0.97	76.8
Hand-eye coordination improved after repeated practice	4.28	0.92	80.4
Reduced dependence on direct supervision	3.98	1.07	68.8
Improved ability to anticipate intraoperative complications	3.96	1.05	66.1
Skills transferable to real surgical practice	4.15	0.97	78.6
Easier transition to the operating theater	4.21	0.88	81.2
Reduced errors during early clinical practice	4.3	0.92	80.4
Reduced fear of making mistakes on real patients	4.24	0.94	81.2
Improved mental preparedness before procedures	4.29	0.83	83.9
Promotes a safe environment for learning from errors	4.4	0.76	84.8
Improved confidence and self-esteem as a surgeon	4.29	0.99	79.5

**Figure 1:**
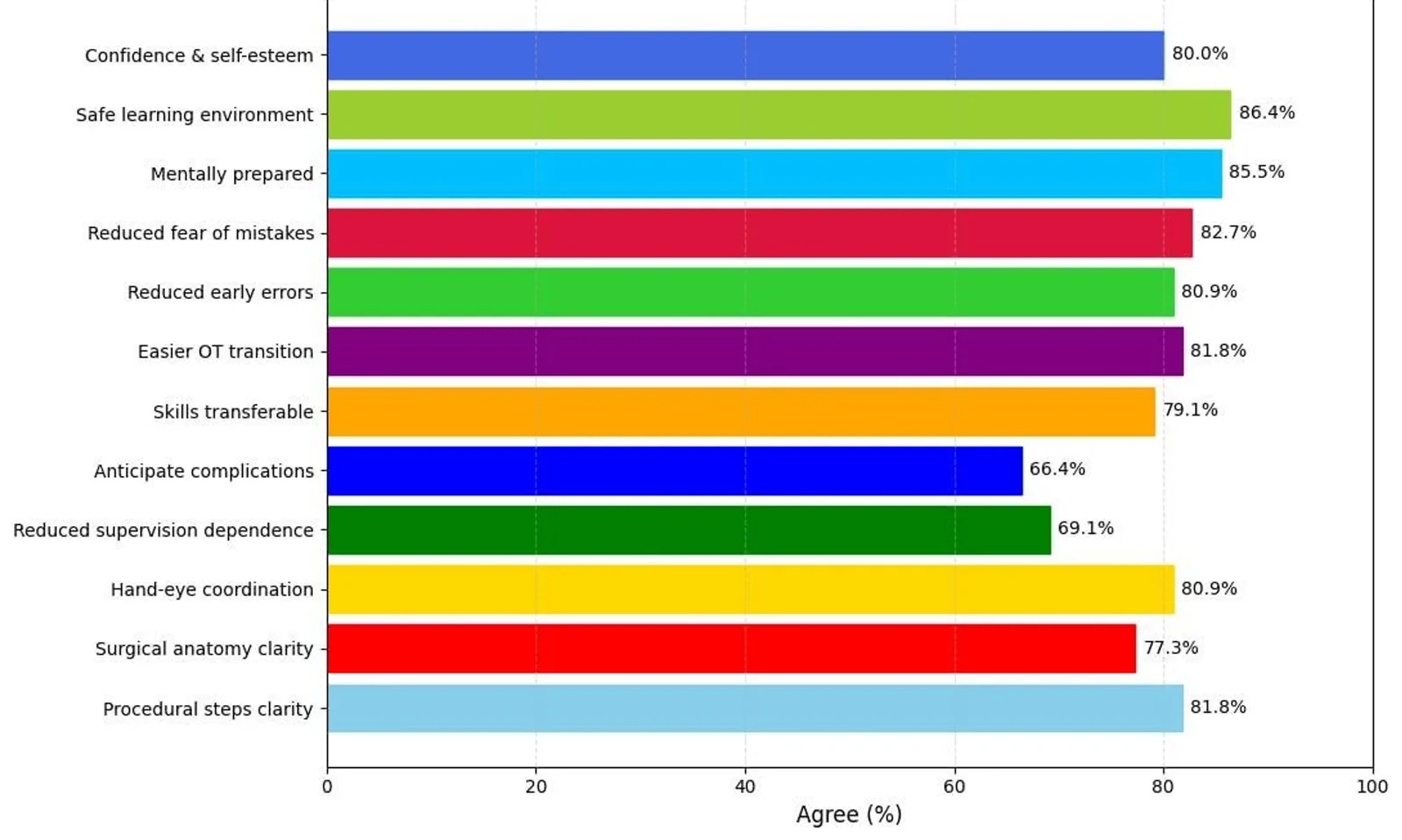
Composite domain scores for perceived educational impact of simulation-based surgical training.

Responses related to cognitive preparedness demonstrated greater variability compared with other assessed domains, reflected by wider dispersion and larger error bars, indicating increased heterogeneity in participant-reported mental readiness following simulation-based training, as shown in Figure 2.

**Figure 2:**
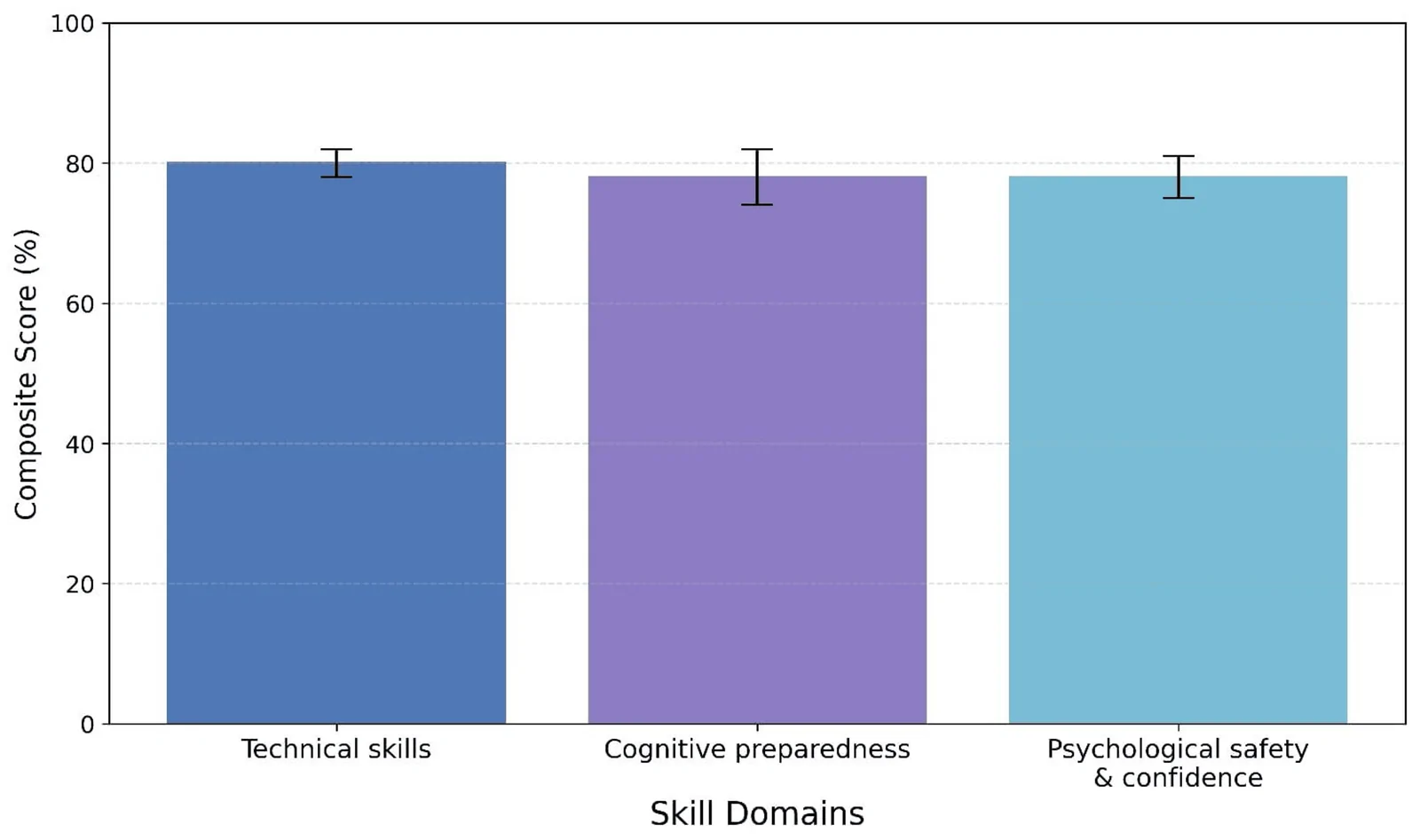
Mean composite scores for each skill domain, with error bars representing standard deviation (n = 112).

Attitudes toward integration and assessment

Beyond the perceived educational benefits, there was strong support for integrating simulation into formal surgical training and assessment.

Early introduction of simulation-based training in surgical education was supported by 100 respondents (89.3%). The incorporation of simulation into formal undergraduate assessments was endorsed by 92 (82.1%), while 96 (85.7%) supported its use in postgraduate assessment frameworks.

Most participants (n = 94, 83.9%) favored the use of simulation as a complementary modality alongside, but not as a replacement for real operating theater exposure.

Perceived limitations and barriers

Concerns regarding inadequate supervision during simulation were expressed by 85 individuals (75.9%). Limitations in perceived realism were identified by 62 (55.4%), while 52 (46.4%) raised concerns that simulation could be misused as a substitute for real-life surgical experience.

Additionally, 66 (58.9%) believed that skills acquired through simulation may be perceived as inferior to those developed in real operating theater environments. Resource constraints emerged as a major barrier, with 90 respondents (80.4%) reporting insufficient availability of simulation facilities and training resources (Table 4, Figure 3).

**Table 4:** Mean Composite Scores for Each Skill Domain, With Error Bars Representing Standard Deviation (n = 112).

Item	Mean	SD	Agree (%)
Lack of constant supervision during simulation sessions	4.2	0.87	75.9
Limited reflection of real surgical scenarios	3.64	1.1	55.4
Concern regarding replacement of real-life surgical experience	3.45	1.22	46.4
Lack of adequate resources for simulation learning	4.33	0.81	80.4

**Figure 3:**
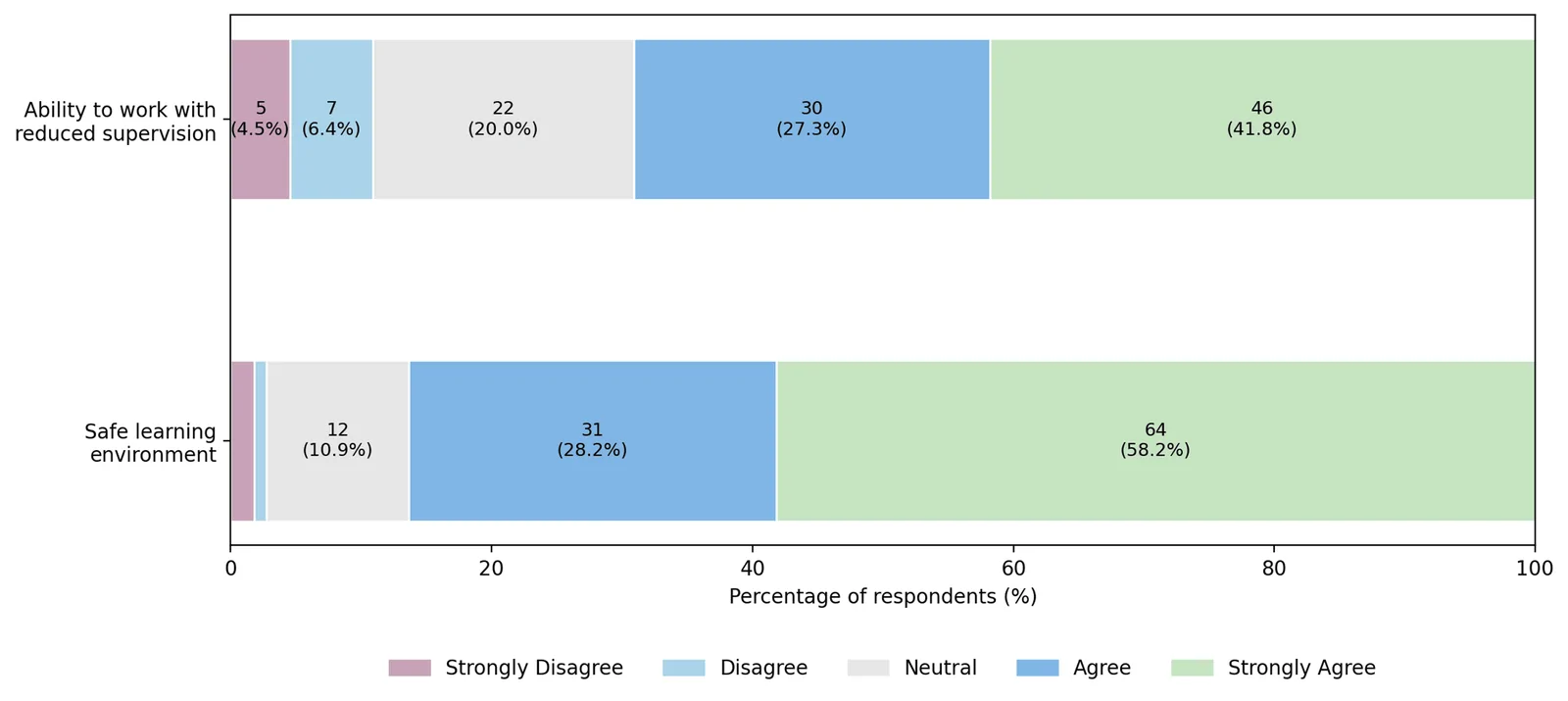
Distribution of participant responses to statements related to supervision and the learning environment.

## Discussion

This international cross-sectional study evaluated the use and perceived educational impact of simulation-based surgical training across multiple countries and stages of surgical education. Among the 112 participants, 59 individuals (52.7%) reported prior exposure to simulation-based training, indicating that although simulation is increasingly recognized as a valuable educational tool, its implementation remains moderate across many healthcare systems. Despite this limited exposure, participants who had experience with simulation reported substantial perceived benefits, particularly in the development of technical skills, procedural understanding, and preparedness for clinical practice. These findings suggest that even relatively limited exposure to simulation may contribute meaningfully to surgical training while also highlighting the need for broader integration of simulation-based education.

In the present study, simulation training was most commonly delivered through bench-top trainers and cadaveric models, while familiarity with virtual reality and hybrid simulation technologies was comparatively lower. This pattern likely reflects differences in institutional resources and infrastructure across participating countries. Importantly, the predominance of low-cost simulation modalities in this study suggests that meaningful educational outcomes do not necessarily require high-fidelity or technologically advanced simulation platforms. Previous literature has similarly demonstrated that a wide range of simulation modalities - including physical task trainers, cadaveric models, and computer-based simulations - can effectively support the acquisition of surgical skills and clinical competencies [10]. Furthermore, studies conducted in low- and middle-income settings have shown that simulation training can significantly improve procedural performance even when implemented using relatively simple and low-cost training models [11].

Suturing was the most frequently practiced technical skill among participants, indicating that simulation is currently used primarily for foundational surgical skills training rather than advanced procedural instruction. This observation aligns with prior research suggesting that simulation-based training is particularly valuable during the early stages of surgical education, where it allows trainees to develop fundamental psychomotor skills before performing procedures in real clinical environments [12]. Developing these basic competencies in a simulated setting may help reduce the early learning curve associated with surgical training and improve trainee confidence before operating on patients.

Participants in this study consistently reported improvements across several key domains of surgical skill development, including procedural understanding, hand-eye coordination, and confidence during surgical tasks (Table 3). Simulation was also widely reported to provide a safe learning environment and to reduce fear of making mistakes during procedures. These findings reinforce the role of simulation as a psychologically safe environment where trainees can practice and refine skills without compromising patient safety. Similar perceptions have been reported in previous studies of surgical trainees, in which the majority of residents reported that simulation improves operative skills and facilitates the transfer of those skills to real surgical settings [12,13].

Composite domain analysis further supported these observations. The highest perceived improvement was observed in technical skill development (mean score ≈80%), followed by cognitive preparedness (≈78.5%) and psychological safety and confidence (≈78%). The slightly lower score observed for psychological safety and confidence may reflect differences in prior simulation exposure, clinical experience, or training stage among participants. While simulation was perceived to be effective for improving technical and psychomotor skills, the development of psychological readiness and self-confidence may require sustained and repeated simulation exposure over time, in addition to real clinical experience. Previous studies have similarly shown that simulation-based training can improve both technical and non-technical competencies, including decision-making, communication, and teamwork in surgical environments [10].

Beyond technical skill development, simulation was widely perceived as a confidence-building educational strategy. A substantial proportion of participants reported that simulation helped reduce anxiety related to procedural errors and improved psychological readiness for performing surgical tasks. These findings align with existing evidence indicating that simulation environments promote psychological safety by allowing trainees to make and correct errors without risk to patients [13]. Randomized controlled trials have further demonstrated that simulation-based training can lead to measurable improvements in surgical performance, including higher proficiency scores and reduced complication rates during real operative procedures [14]. Together, these findings suggest that simulation-based education can serve as an effective bridge between theoretical knowledge and real clinical practice.

Despite these benefits, several barriers to the broader implementation of simulation training were identified. Participants reported inconsistent access to simulation facilities, equipment, and trained instructors, particularly in resource-limited settings. These findings are consistent with previous multinational studies that have identified cost, infrastructure limitations, and lack of trained faculty as major barriers to the implementation of simulation-based surgical education programs. Similarly, large surveys of surgical trainees in the United Kingdom and Ireland have shown that although nearly all trainees recognize the importance of simulation training, many report limited access to structured simulation programs within their institutions [13]. Addressing these structural barriers will be essential for expanding the role of simulation in surgical training programs.

Participants in this study also strongly supported the integration of simulation into formal undergraduate and postgraduate surgical training curricula, while emphasizing that simulation should complement rather than replace operative experience. This perspective reflects the evolving paradigm of surgical education, in which simulation is increasingly viewed as an adjunct to traditional apprenticeship-based learning. By allowing trainees to develop baseline competencies before performing procedures on patients, simulation-based education may improve both training efficiency and patient safety [10].

Several limitations should be considered when interpreting the findings of this study. First, the use of convenience sampling and self-reported survey data may introduce response bias. A formal response rate could not be calculated, as the questionnaire was distributed electronically without a fixed, trackable list of invited participants. The distribution of respondents across the 49 participating hospitals was not systematically recorded, limiting assessment of site-level representativeness. The pilot testing of the questionnaire was limited to an assessment of clarity and content relevance; formal psychometric evaluation of validity or reliability (e.g., Cronbach's alpha) was not performed. Second, the cross-sectional design limits the ability to establish causal relationships between simulation exposure and objective improvements in surgical performance. Third, the study relied on participants' perceptions of training effectiveness rather than objective performance metrics such as procedural accuracy or operative outcomes. Finally, differences in institutional training structures across the participating countries may have influenced both exposure to simulation and perceptions of its educational value. The sample size, while adequate for descriptive analysis, is relatively modest for a multinational study and was heavily skewed toward participants based in Bangladesh, limiting the generalizability of findings to other national contexts. Potential differences in simulation training curricula, resources, and methods employed across the participating countries were not statistically assessed or controlled for in this analysis. Despite these limitations, this study provides valuable insights into the current use and perceived impact of simulation-based surgical training across multiple countries and levels of training. The inclusion of participants from diverse healthcare systems enhances the generalizability of the findings and highlights both the educational benefits and implementation challenges associated with simulation training.

Overall, the results support simulation-based training as an important component of modern surgical education that is perceived by participants to contribute to the development of technical competence, confidence, and clinical preparedness; as a self-reported, cross-sectional study, these findings reflect participants' perceptions rather than objectively measured outcomes. Future research should focus on longitudinal evaluation of simulation training programs, incorporation of objective performance assessments, and investigation of patient-related outcomes following simulation-based training. Such studies will be essential for further defining the role of simulation in surgical education and for guiding the development of evidence-based simulation curricula worldwide.

## Conclusions

This multinational survey of 112 doctors and trainees across 49 hospitals in five countries demonstrates a strong and consistent perception of improved technical competence, procedural confidence, and psychological readiness for surgical practice associated with simulation-based training, while also easing the anxiety associated with early operative responsibility. Respondents strongly supported earlier integration of simulation into surgical curricula and its formal incorporation into undergraduate and postgraduate assessment, while emphasizing that simulation should complement, rather than replace, real operative experience.

At the same time, resource constraints and inconsistent supervision remain significant obstacles to wider implementation, particularly in lower-resource settings. These findings support targeted efforts to expand access to adequately resourced and supervised simulation programs, alongside future research using objective performance measures and longitudinal follow-up to clarify the impact of simulation on clinical outcomes.

## Appendices

**Table 5:** Questionnaire: Participant Characteristics and Simulation Exposure (Q1–10)

No.		Response Option
1. Current role in the medical field		Undergraduate medical student
		Intern
		Medical officer
		Postgraduate trainee
		Consultant
		Others
2. Primary surgical specialty of interest		General surgery
		Ophthalmology
		Orthopedics
		Anesthesia
		Neurosurgery
		Cardiothoracic surgery
		Burn and plastic surgery
		Pediatric surgery
		Others
3. Years of training in surgery		<1 year
		1–2 years
		3–5 years
		>5 years
4. Current Hospital/College of employment or training		Free text
5. Do you have any previous exposure in surgical simulation based learning?		Yes
		No
6. What are the types of simulation modalities that you have practiced on? (Pick all that apply)		Bench Top Model
		Live Animal Models
		Cadaveric Models
		Virtual Reality Models
		Hybrid Simulators
7. Average duration of simulation sessions		< 1 hour
		1–2 hours
		> 2 hours
8. Frequency of simulation sessions		Once a year
		Few times a year
		Few times a month
		Few times a week
		Daily
9. What are the surgical skills you have practiced through simulation based learning? (Pick all that apply)		Suture
		Excisions
		Biopsy techniques
		Flap surgery
		Swelling excisions
		Injections
		CPR
		Endoscopy
		Laparoscopy
		Others
10. Simulation sessions were mainly used for:		Skill acquisition
		Assessment
		Revision before clinical exposure
		Emergency preparedness

**Table 6:** Response Options for Likert-Scale Statements

Scale value	Response Option
1	Strongly Disagree
2	Disagree
3	Neutral
4	Agree
5	Strongly Agree

**Table 7:** Questionnaire: Perceived Impact, Attitudes, and Limitations of Simulation-Based Training (Q11–31)

No.		Response Option
1. Current role in the medical field		Undergraduate medical student
		Intern
		Medical officer
		Postgraduate trainee
		Consultant
		Others
2. Primary surgical specialty of interest		General surgery
		Ophthalmology
		Orthopedics
		Anesthesia
		Neurosurgery
		Cardiothoracic surgery
		Burn and plastic surgery
		Pediatric surgery
		Others
3. Years of training in surgery		<1 year
		1–2 years
		3–5 years
		>5 years
4. Current Hospital/College of employment or training		Free text
5. Do you have any previous exposure in surgical simulation based learning?		Yes
		No
6. What are the types of simulation modalities that you have practiced on? (Pick all that apply)		Bench Top Model
		Live Animal Models
		Cadaveric Models
		Virtual Reality Models
		Hybrid Simulators
7. Average duration of simulation sessions		< 1 hour
		1–2 hours
		> 2 hours
8. Frequency of simulation sessions		Once a year
		Few times a year
		Few times a month
		Few times a week
		Daily
9. What are the surgical skills you have practiced through simulation based learning? (Pick all that apply)		Suture
		Excisions
		Biopsy techniques
		Flap surgery
		Swelling excisions
		Injections
		CPR
		Endoscopy
		Laparoscopy
		Others
10. Simulation sessions were mainly used for:		Skill acquisition
		Assessment
		Revision before clinical exposure
		Emergency preparedness
